# The Association of Statin Use after Cancer Diagnosis with Survival in Pancreatic Cancer Patients: A SEER-Medicare Analysis

**DOI:** 10.1371/journal.pone.0121783

**Published:** 2015-04-01

**Authors:** Christie Y. Jeon, Stephen J. Pandol, Bechien Wu, Galen Cook-Wiens, Roberta A. Gottlieb, Noel Bairey Merz, Marc T. Goodman

**Affiliations:** 1 Samuel Oschin Comprehensive Cancer Institute, Cedars-Sinai Medical Center, Los Angeles, CA, United States of America; 2 Department of Epidemiology, Fielding School of Public Health, University of California Los Angeles, Los Angeles, CA, United States of America; 3 Department of Medicine, Cedars-Sinai Medical Center, Los Angeles, CA, United States of America; 4 Department of Veterans Affairs, Los Angeles, CA, United States of America; 5 Department of Gastroenterology, Kaiser Permanente Los Angeles Medical Center, Los Angeles, CA, United States of America; 6 Biostatistics and Bioinformatics Research Center, Cedars-Sinai Medical Center, Los Angeles, CA, United States of America; 7 Barbra Streisand Women’s Heart Center, Heart Institute of Cedars-Sinai, Los Angeles, CA, United States of America; 8 Department of Biomedical Sciences, Cedars-Sinai Medical Center, Los Angeles, CA, United States of America; Leibniz Institute for Age Research, GERMANY

## Abstract

**Background:**

Pancreatic cancer has poor prognosis and existing interventions provide a modest benefit. Statin has anti-cancer properties that might enhance survival in pancreatic cancer patients. We sought to determine whether statin treatment after cancer diagnosis is associated with longer survival in those with pancreatic ductal adenocarcinoma (PDAC).

**Methods:**

We analyzed data on 7813 elderly patients with PDAC using the linked Surveillance, Epidemiology, and End Results (SEER) - Medicare claims files. Information on the type, intensity and duration of statin use after cancer diagnosis was extracted from Medicare Part D. We treated statin as a time-dependent variable in a Cox regression model to determine the association with overall survival adjusting for follow-up, age, sex, race, neighborhood income, stage, grade, tumor size, pancreatectomy, chemotherapy, radiation, obesity, dyslipidemia, diabetes, chronic pancreatitis and chronic obstructive pulmonary disease (COPD).

**Results:**

Overall, statin use after cancer diagnosis was not significantly associated with survival when all PDAC patients were considered (HR = 0.94, 95%CI 0.89, 1.01). However, statin use after cancer diagnosis *was associated with a 21% reduced hazard of death* (Hazard ratio = 0.79, 95% confidence interval (CI) 0.67, 0.93) in those with grade I or II PDAC and to a similar extent in those who had undergone a pancreatectomy, in those with chronic pancreatitis and in those who had not been treated with statin prior to cancer diagnosis.

**Conclusions:**

We found that statin treatment after cancer diagnosis is associated with enhanced survival in patients with low-grade, resectable PDAC.

## Introduction

The prognosis for persons with pancreatic cancer remains poor, with an estimated 5-year survival probability of 6%.[1] With the recent increase in incidence [1], pancreatic cancer is projected to be the 2^nd^ leading cause of cancer death in the U.S. by year 2020 [2].

Existing interventions for pancreatic cancer, including surgical resection and gemcitabine treatment are limited in scope and provide only a modest benefit [3]; and there remains a need for alternative therapeutic agents that improve pancreatic cancer outcomes. Mechanistic studies demonstrate that HMG-CoA reductase inhibitors, or statins, not only mitigate elevated cholesterol levels, but also appear to have anti-cancer properties through inhibition of post-translational modification of key proteins involved in tumor proliferation and metastasis [4]. In line with the molecular effects, an observational study of 250 pancreatic cancer patients found that statin treatment is associated with improved survival among diabetics [5], although a clinical trial of simvastatin in 114 gemcitabine-treated patients with advanced pancreatic cancer observed no effect of a 3-week statin regimen on survival [6]. Both of these studies were limited in sample size and not population-based. Furthermore, questions regarding the variability in effect by statin type and intensity, as well as cancer stage and interventions remain unexplored.

Given the dismal prognosis for pancreatic cancer patients and the widely accepted tolerability of statins, we examined the hypothesis that statin treatment may provide a survival advantage among an elderly population with pancreatic adenocarcinoma using the linked Surveillance, Epidemiology, and End Results (SEER)—Medicare claims files. Special advantages of these linked data include the provision of a nationally representative sample of cancer patients in the U.S., as well as a comprehensive record of type, timing and intensity of statin prescriptions filled by the cancer patients.

## Methods

### Study population

**Fig 1:**
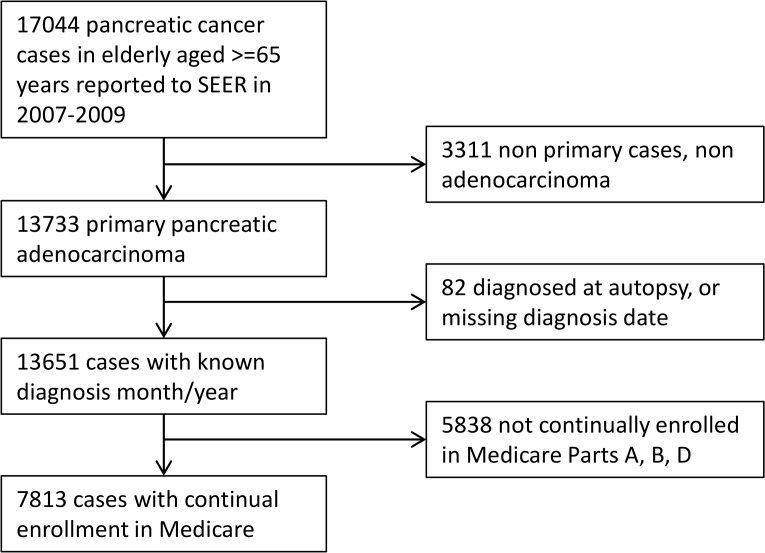
Selection of Medicare patients diagnosed with primary pancreatic adenocarcinoma in 2007–2009.

We conducted a retrospective cohort study of elderly pancreatic cancer patients represented in the SEER-Medicare database. SEER is a national program of 18 regional or state-wide cancer registries in the U.S. Since 1991, the Centers for Medicaid and Medicare Services (CMS) has partnered with SEER to link the cancer registries and claims-based data in Medicare-enrolled populations to facilitate health services research [7]. Starting in 2007, CMS also began to link data from Medicare Part D, a newly implemented insurance program for prescription drug coverage. As of 2014, SEER Program data were available for patients diagnosed with cancer through 2009 and prescription data on Medicare Part D enrollees were available starting in 2007; therefore, patients with primary pancreatic adenocarcinoma diagnosed from 2007 to 2009 were selected for the analysis. We restricted the study population to patients who were continuously enrolled in Medicare Part D beginning 3 months before cancer diagnosis to death or end of follow-up (December 31st, 2010). This design permitted the investigation of outcomes among patients exposed to statins following cancer diagnosis as well as the potential modifying effect of statin exposure for up to 3 months before cancer diagnosis. As we wished to capture information on comorbidities present prior to cancer diagnosis and cancer-related procedures present in inpatient and outpatient claims files, we further restricted the study population to persons who were continuously enrolled in Medicare Parts A and B from 12 months before their cancer diagnosis until death or end of follow-up. A total of 17044 pancreatic cancer cases among elderly aged 65 years or older were reported to SEER in 2007–2009. Excluding patients who did not have primary pancreatic adenocarcinoma of malignant form, patients who were diagnosed at autopsy or had unknown time of diagnosis, and those without continual enrollment in Medicare A, B and D, 7813 patients remained and made up the final analytic population for the present study. (Fig. 1)

## Data

### SEER registries data

Among patients with ‘pancreas’ as the primary site of cancer in the SEER registries, adenocarcinoma types were specifically selected for using ICD-O-3 histology codes: 8000, 8010, 8020, 8021, 8022, 8140, 8141, 8211, 8230, 8500, 8521, 8050, 8260, 8441, 8450, 8453, 8470, 8471, 8472, 8473, 8480, 8481, 8503. Patients were characterized by demographic factors including, age, sex, race, and neighborhood income, and clinical data, including grade, stage and tumor size at diagnosis. Only cases confirmed by microscopy, laboratory markers, radiography, and/or direct visual inspection were selected for the analyses. The outcome of interest was overall survival since the time of cancer diagnosis to death or December 31^st^, 2010, the last date of available Medicare claims data.

### Medicare claims data

Exposure to statins after cancer diagnosis was assessed both as a binary variable (ever use after cancer diagnosis vs. never use), and as an ordinal variable (high, moderate, low intensity vs. never use). High, moderate and low intensity statin therapy followed the categorizations set forth by the American Heart Association and the American College of Cardiologists [8]. We also investigated the associations of statin use with survival by statin potency and lipophilicity: high potency statins (atorvastatin, rosuvastatin, simvastatin) or low potency statins (fluvastatin, lovastatin, pravastatin), lipophilic statins (atorvastatin, fluvastatin, lovastatin, simvastatin) or hydrophilic statins (pravastatin, rosuvastatin).

Surgical resection of the pancreas was determined through the Medicare inpatient files using ICD9 procedure codes: 52.6, 52.7, 52.51, 52.52, 52.53, 52.59. Dyslipidemia, diabetes or impaired glucose tolerance (IGT), obesity, chronic pancreatitis, and chronic obstructive pulmonary disease (COPD) were extracted through the Medicare claims files from inpatient and outpatient records and were defined using the following ICD9 diagnostic codes: Diabetes mellitus / Impaired glucose tolerance: 250.xx, 790.2, 790.21, 790.22, 790.29, Overweight, Obesity: 278.0, 278.1, 278.00, 278.01, 278.02, 783.1, v77.8, Dyslipidemia: 272.0, 272.1, 272.2, 272.4, 272.5, 272.9, COPD: 491.0, 491.1, 491.20, 491.21, 491.8, 491.9, 492.0, 492.8, 494, 494.0, 494.1, 496, chronic pancreatitis: 577.1. Due to the lack of data on smoking, an established risk factor for PDAC, we included COPD as a proxy variable for smoking-related diseases. We also obtained information regarding chemotherapy and radiation treatment through the Medicare files using commonly used ICD9 and Current Procedural Terminology codes [9].

### Statistical analyses

The distributions of demographic, pathologic, and clinical characteristics were compared between statin users and non-users by Chi-squared analyses. We estimated the median survival times from diagnosis to death by statin use using Kaplan-Meier analyses, testing for differences by the log-rank test. We examined the relationship between statin use and survival in PDAC patients through several Cox regression approaches. First we modeled the relationship between ever use of statin after cancer diagnosis as a non-time-dependent variable, i.e. patients who ever used statin after cancer diagnosis were classified as statin users as that from the time of cancer diagnosis. Next, we analyzed statin as a time-dependent covariate given the potential for immortal time bias, that is, the extended length of survival time between cancer diagnosis and statin exposure during which death could not have occurred [10]. To prevent this bias from affecting our results we classified 1) patients who were already on statin as statin users from the time of cancer diagnosis and 2) patients who were not on statin at the time of cancer diagnosis, but filled a statin prescription after cancer diagnosis as non-users until they filled the statin prescription. We then evaluated the independent association of statin use after cancer diagnosis and survival by sequentially adding the following potential confounders: 1) age, sex, race, neighborhood income, 2) stage, grade and tumor size, 3) resection radiation and chemotherapy, 4) obesity, dyslipidemia, diabetes/IGT, chronic pancreatitis and COPD. Receipt of a pancreatectomy, chemotherapy or radiation were also incorporated as time-dependent covariates when the intervention was first indicated on or after cancer diagnosis based on dates reported in Medicare claims files. We considered chemotherapy and radiation administered within 6 months of the pancreatic cancer diagnosis as relevant interventions for the primary cancer. In addition, to evaluate the influence of reverse causation, that is, the impact of end-of-life decisions on statin use, we conducted a multivariable model in which time-dependent statin exposure was lagged by 2 months prior to the time of death and in a contemporaneous period in those who were alive. In this analysis, we restricted the population to those with at least 2 months survival. We performed stratified analyses by selected variables to identify modifiers of the statin effect: age group, summary stage, grade, tumor size, resection, chemotherapy, dyslipidemia and diabetes. Heterogeneity in effect was tested by an interaction term with the time-dependent statin variable in the multivariable models. The multivariable models examining the interactions were based on the analysis that incorporates the lagged time dependent statin variable. All statistical analyses were conducted in SAS Version 9.3 (SAS Institute in Cary, NC).

## Results

Of 7813 primary pancreatic adenocarcinoma cases, 20% were 85 years or older, 41% were male, and 78% were of White race (Table 1). Cancer was staged as local or regional in 36% of the population. Surgical resection was performed on 11% of the cases, and 21% of the cases had chemotherapy. The prevalence of surgical management and chemotherapy are comparable to previous publication on pancreatic cancer patients represented in SEER-Medicare [11]. Thirteen percent of the cases had evidence of ICD9 diagnoses of overweight/obesity, 67% dyslipidemia, and 48% diabetes. A majority of the study population (93%) died in the follow-up period (median follow-up 3.1 months, interquartile range [IQR]: 1.3, 8.6).

**Table 1:** Distribution of demographic, clinical and comorbid characteristics by statin treatment after diagnosis with pancreatic adenocarcinoma.

Variables	Category	Total (n = 7813)		Not exposed to statin after cancer diagnosis n = 5357 (69%)		Exposed to statin after cancer diagnosis n = 2456 (31%)		P-value
		**n**	**column %**	**n**	**column %**	**n**	**column %**	
Age								<0.0001
	65–74	2932	(38)	1939	(36)	993	(40)	
	75–84	3311	(42)	2230	(42)	1081	(44)	
	85+	1570	(20)	1188	(22)	382	(16)	
Sex								0.79
	Male	3212	(41)	2197	(41)	1015	(41)	
	Female	4601	(59)	3160	(59)	1441	(59)	
Race								0.001
	White	6121	(78)	4207	(79)	1914	(78)	
	Black	818	(10)	590	(11)	228	(9)	
	Other	874	(11)	560	(10)	314	(13)	
Neighborhood median income								0.0002
	<$35,000 OR unknown*	1986	(25)	1411	(26)	575	(23)	
	$35,000-$49,999	2508	(32)	1760	(33)	748	(30)	
	$50,000-$74,999	2279	(29)	1517	(28)	762	(31)	
	$75,000 +	1040	(13)	669	(12)	371	(15)	
Summary stage								<0.0001
	Localized/Regional	2832	(36)	1837	(34)	995	(41)	
	Distant	4331	(55)	3039	(57)	1292	(53)	
	Stage unavailable	650	(8)	481	(9)	169	(7)	
Grade								<0.0001
	1 or 2	1150	(15)	718	(13)	432	(18)	
	3 or 4	954	(12)	640	(12)	314	(13)	
	Unknown	5709	(73)	3999	(75)	1710	(70)	
Tumor size								<0.0001
	<5cm	3986	(51)	2601	(49)	1385	(56)	
	> = 5cm	1471	(19)	1030	(19)	441	(18)	
	Unknown	2356	(30)	1726	(32)	630	(26)	
Resection		842	(11)	500	(9)	342	(14)	<0.0001
Chemotherapy		1635	(21)	998	(19)	637	(26)	<0.0001
Radiation		606	(8)	368	(7)	238	(10)	<0.0001
Obesity		990	(13)	647	(12)	343	(14)	0.02
Chronic pancreatitis		497	(6)	347	(6)	150	(6)	0.53
COPD		2947	(38)	2067	(39)	880	(36)	0.02
Dyslipidemia		5259	(67)	3389	(63)	1870	(76)	<0.0001
Diabetes/IGT		3729	(48)	2370	(44)	1359	(55)	<0.0001

*Unknown income group comprise less than 2% in this category.

Numbers are not reported due to small N.

Statin treatment after cancer diagnosis was reported in 31% of the patient population (Table 1). Patients who used statin after cancer diagnosis were younger, and more likely to have local/regional disease, lower grade, and smaller tumor size. Statin users were also more likely to receive surgical resection, chemotherapy, and radiotherapy; and were more likely to be diagnosed with obesity, dyslipidemia, and diabetes. Among PDAC patients who used statin after cancer diagnosis, simvastatin was most commonly prescribed (48%), followed by atorvastatin (25%), and lovastatin (18%) (Table 2). Most patients were on lipophilic statins, of high potency and low-to-moderate dose. The proportion of follow-up days on statin did not vary by statin intensity (57% in low, 56% in moderate, and 55% in high intensity statin users; data not shown). The median survival time was significantly longer among patients who used statin after cancer diagnosis (4.7 months, interquartile range: 1.9–11.7) compared to those who were never prescribed a statin after cancer diagnosis (2.4 months, interquartile range: 1.1–7.3).

**Table 2:** Description of statin use after cancer diagnosis by name, lipophilicity, potencity, intensity, duration, consistency of use (n = 2456).

Variables	Category	n	column %
Statin name*			
	Atorvastatin	605	(25)
	Fluvastatin	16	(1)
	Lovastatin	452	(18)
	Pravastatin	171	(7)
	Rosuvastatin	116	(5)
	Simvastatin	1184	(48)
Statin lipophilicity*			
	Hydrophilic	284	(12)
	Lipophilic	2199	(90)
Statin potency*			
	High	1864	(76)
	Low	639	(26)
Statin intensity			
	Low	400	(16)
	Moderate	1663	(68)
	High	393	(16)
Statin use pre-cancer diagnosis			
	No	158	(6)
	Yes	2298	(94)

* Categories are not mutually exclusive for these variables

Table 3 presents the results of the association of survival with statin use after cancer diagnosis in PDAC patients from several modeling approaches. The estimated HR of 0.76 for a *time-dependent* statin variable from model 2 was weaker compared to the HR of 0.69 estimated for a *non-time-dependent* statin variable in model 1, showing that the assumptions for the non-time-dependent effect of statin, including immortal time bias, had exaggerated the statin-survival association in model 1 (Table 3). The association of statin use after PDAC diagnosis and survival remained significant after adjusting for all potential confounders considered (HR = 0.79, 95% CI 0.75, 0.83) (Table 3). When we evaluated the association of statin exposure lagged by 2 months with survival, an approach that is less prone to bias from reverse causation, we observed an attenuated and non-significant association between statin use and survival (HR = 0.94, 95% CI 0.75, 1.01). This analysis, which was limited to those who survived for at least 2 months (n = 4705), showed that end of life variation in statin prescription fills had biased our results.

**Table 3:** Relative hazard of death for statin use after cancer diagnosis vs. no statin use (n = 7813).

Model	HR (95%)	P-value
Model 1: Unadjusted model using statin as a *non-time-dependent variable*	0.69 (0.66, 0.72)	<0.0001
Model 2: Unadjusted model using statin as a time-dependent variable (removes immortal time bias)	0.76 (0.72, 0.80)	<0.0001
Model 3: Model 2 + age, sex, race, neighborhood income adjustment	0.79 (0.75, 0.83)	<0.0001
Model 4: Model 3 + stage, grade, tumor size	0.80 (0.76, 0.84)	<0.0001
Model 5: Model 4 + resection, radiation and chemotherapy	0.80 (0.76, 0.84)	<0.0001
Model 6: Model 5 + obesity, dyslipidemia, diabetes/IGT, chronic pancreatitis and COPD	0.79 (0.75, 0.93)	<0.0001
Model 7: Modeling statin as a time-dependent variable that lags by 2 months (removes reverse causation, restricts population to >2 month survivors)	0.94 (0.89, 1.01)	0.08

HR = hazard ratio for statin use; IGT = impaired glucose tolerance, COPD = chronic obstructive pulmonary disease

Important heterogeneity in the association between statin use after cancer diagnosis and survival in PDAC patients was observed in stratified analyses. We found an inverse association of statin use after cancer diagnosis with survival in those with grade I or II cancer (HR = 0.79, 95%CI 0.67, 0.93), but not in those with grade III or IV cancers (HR = 1.07, 95%CI 0.89, 1.29; p for interaction = 0.02). Furthermore, patients with chronic pancreatitis were more likely to experience a survival advantage associated with statin use cancer diagnosis (HR = 0.75, 95%CI 0.56, 0.99) compared to those without chronic pancreatitis (HR = 0.96, 95%CI 0.90, 1.03; p for interaction = 0.03). Statin use after cancer diagnosis was also associated with survival in those who underwent a pancreatectomy (HR = 0.80, 95%CI 0.66, 0.97) versus no surgery, in those diagnosed with COPD (HR = 0.89, 95%CI 0.80, 1.00) versus no COPD, and in those who had not been treated with statin prior to cancer diagnosis (HR = 0.80, 95%CI 0.63, 1.00) versus those with previous treatment with statin. The differenced in HRs by the latter three potential effect modifiers were marginally significant (Table 4). No significant difference in the association of statin use with survival was observed by testing for interaction with age, summary stage, tumor size, chemotherapy, dyslipidemia, or diabetes.

**Table 4:** Multivariable-adjusted association between statin use after diagnosis with pancreatic cancer and overall survival in *substrata of covariates*.

Variables	Category	Adjusted HR (95% CI) for death comparing statin use vs. non-use	p-value	P-value for interaction
Age				0.86
	65–74	0.94 (0.85, 1.04)	0.23	
	75–84	0.93 (0.84, 1.03)	0.15	
	85+	0.96 (0.81, 1.15)	0.68	
Summary stage				0.34
	Localized/Regional	0.91 (0.82, 1.00)	0.051	
	Distant	0.99 (0.90, 1.08)	0.75	
**Grade**				**0.02**
	**1 or 2**	**0.79 (0.67, 0.93)**	**0.004**	
	**3 or 4**	**1.07 (0.89, 1.29)**	**0.45**	
Tumor size				0.38
	<5cm	0.94 (0.86, 1.02)	0.14	
	≥5cm	0.88 (0.75, 1.03)	0.11	
**Resection**				**0.054**
	**Yes**	**0.80 (0.66, 0.97)**	**0.02**	
	**No**	**0.96 (0.90, 1.03)**	**0.26**	
Chemotherapy				0.72
	Yes	0.93 (0.82, 1.04)	0.21	
	No	0.96 (0.88, 1.03)	0.25	
Dyslipidemia				0.52
	Yes	0.95 (0.88, 1.03)	0.24	
	No	0.95 (0.84, 1.07)	0.41	
Diabetes/IGT				0.99
	Yes	0.92 (0.84, 1.02)	0.1	
	No	0.95 (0.97, 1.04)	0.25	
**Chronic pancreatitis**				**0.03**
	**Yes**	**0.75 (0.56, 0.99)**	**0.04**	
	**No**	**0.96 (0.90, 1.03)**	**0.15**	
**COPD**				**0.07**
	**Yes**	**0.89 (0.80, 1.00)**	**0.046**	
	**No**	**0.98 (0.91, 1.06)**	**0.66**	
**Statin use pre cancer diagnosis**				**0.11**
	**Yes**	**0.97 (0.84, 1.11)**	**0.65**	
** **	**No**	**0.80 (0.63, 1.00)**	**0.048**	** **
.				

Note: Stratified analysis in which statistically significant associations of statin use after cancer diagnosis with survival are highlighted in **bold** type

When each type of statin used after cancer diagnosis was investigated for independent effects in the same model, only simvastatin treatment was associated with a significantly lower hazard of death compared to no statin treatment (HR = 0.91, 95%CI 0.84, 0.99) (fluvastatin was not considered due to small cell size (n = 16)) (Table 5). When separated by lipophilicity, neither hydrophilic nor lipophilic statin was associated with survival. When separated by potency, those who were on high potency statin experienced significantly decreased hazard of death (HR = 0.93, 95% 0.86, 1.00). Increasing intensity of statin dose was not correlated with stronger inverse associations. Rather, those treated with low dose statin were more likely to show a benefit by statin (HR = 0.85, 95%CI 0.75, 0.97) (Table 5).

**Table 5:** Multivariate association of survival with name, lipophilicity, potency, intensity of statin use after diagnosis of pancreatic adenocarcinoma.

Variables	Category	adjusted HR (95%) for death	P-value
Statin name			
	Atorvastatin vs. no statin	0.97 (0.87, 1.09)	0.59
	Lovastatin vs. no statin	0.96 (0.85, 1.08)	0.48
	Pravastatin vs. no statin	1.07 (0.88, 1.30)	0.53
	Rosuvastatin vs. no statin	0.98 (0.79, 1.23)	0.87
	Simvastatin vs. no statin	0.91 (0.84, 0.99)	0.03
Statin type			
	Hydrophilic vs. no statin	1.02 (0.87, 1.18)	0.85
	Lipophilic vs. no statin	0.94 (0.88, 1.01)	0.08
Statin potency			
	High potency vs. no statin	0.93 (0.86, 1.00)	0.035
	Low potency vs. no statin	0.99 (0.89, 1.10)	0.8
Statin intensity			
	Low vs. no statin	0.85 (0.75, 0.97)	0.02
	Moderate vs. no statin	0.97 (0.90, 1.04)	0.35
	High vs. no statin	0.97 (0.84, 1.11)	0.65

## Discussion

Our study is the first population-based analysis of the association of statin and pancreatic cancer survival and is the first study to examine how the outcomes vary by specific statin type and intensity of use. Compared to those who did not use statins, the median survival in patients who used statin after cancer diagnosis was increased by 1.3 months overall. Accounting for immortal time between cancer diagnosis and statin treatment, reverse causation, as well as multiple demographic, clinical and comorbid factors, the *overall association* between statin use after cancer diagnosis and survival in PDAC patients was attenuated to a non-significant relationship, although patients on simvastatin, and those on high potency statins (simvastatin, rosuvastatin, atorvastatin) still showed a significant 7–9% reduction in rate of death. We found no dose-response association with greater intensity of statin dose; the use of lower intensity statins showed a modestly stronger association with survival which was not attributable to more consistent use.

While the overall associations on statin use after cancer diagnosis and survival was not significant, important differences in the statin-survival associations by grade, resection, chronic pancreatitis and COPD were found. Statin use after cancer diagnosis showed an association with survival in those who had grade I/II tumors and in those who had underwent a resection, but not in patients with higher grade tumors and those who did not undergo a resection. This finding may have resulted from the ability of statin to prevent the regrowth of well-differentiated pancreatic tumor, which occurs in a majority of those with a resection. We also found that the statin-survival association was significant in those with chronic pancreatitis and people with COPD, but not in those without these conditions. This suggests that statin may have an effect on tumors affected by heavy drinking, a major risk factor for chronic pancreatitis, and smoking, a major risk factor for COPD, although the mechanisms are unknown. Importantly, we found that statin use after cancer diagnosis was associated with survival in those with no exposure to statin prior to cancer diagnosis, but not in those with prior statin exposure. It is conceivable that statin treatment after cancer diagnosis may have a greater impact on statin-naïve tumors that are sensitive to the molecular effects of statin, whereas tumors that arose in patients already receiving statins may have been selected for statin resistance before diagnosis. That statin has a specific impact in those with grade I/II cancers and in those with no previous exposure to statin suggests that there are specific tumor subtypes that likely will respond to statin. Genomic and transcriptomic studies demonstrate that PDAC is a highly heterogeneous cancer type [12], with molecular subtypes that vary in their response to existing therapies [13]. Molecular epidemiologic studies that integrate genetic information at the tumor level with clinical data will help us identify patients who may benefit from statin treatment.

Our study results do not confirm the findings of a Japanese study that showed longer survival with statin use among patients with locally advanced or metastatic pancreatic cancer who underwent chemotherapy [5]. The association of statin with pancreatic cancer survival found in the Japanese study was specific to those with diabetes. We examined the potential interaction between statin use after cancer diagnosis and diabetes, but did not find that the impact on survival varied by diabetes status. The two studies differed importantly in methodology. We adjusted for immortal time bias and reverse causation, while the Japanese study did not consider the influence of these biases. Furthermore, our studies were different in that all patients in our study population were elderly, while half of the Japanese study population was under the age of 66. It is possible that the non-diabetic patients were more similar to diabetic patients in this elderly population than in a younger population who have more beta cells [14]. Similar to our null finding in those treated with chemotherapy and those with distant cancer type, a recent clinical trial of simvastatin in gemcitabine-treated advanced pancreatic cancer patients found no effect of a 3-week regimen of simvastatin on progression-free or overall survival [6].

A wealth of *in vivo* and *in vitro* evidence for the anti-cancer properties of statin supports the therapeutic potential of statin against pancreatic cancer. The inhibition of HMG-CoA reductase by statin results in a decreased production of isoprenoids (e.g. farnesyl pyrophostate), necessary post-translational modifiers of Ras and Rho proteins. Prenylation is required for anchoring Ras to the membrane and subsequent transduction of extracellular growth factor signals to effector pathways essential for cell cycle progression, growth, and survival of cancer cells [4]. For pancreatic cancer, in which oncogenic mutation in K-Ras is common [15], statin could have a particularly pronounced effect through modifying K-Ras activity. Statin is also known to inhibit the membrane association of Rho proteins that are involved in cell adhesion, cell motility, and invasion [4, 16]. In step with these molecular effects, statin treatment has been shown to prevent pancreatic cancer cell invasion *in vitro* and metastasis to the liver *in vivo* [16]. Statin also has been shown to induce autophagy, potentially through depletion of geranylgeranyl diphosphate and activation of AMP kinase [17] and autophagic cell death in some cancer cell lines [18], although the role of autophagy in pancreatic cancer is contradictory [19–21]. A recent animal-model study [21] demonstrated that induction of autophagy in the presence of TP53 mutation limits the growth of tumors. Future studies investigating the heterogeneous effect of statin by common somatic mutations in KRAS, TP53, CDK2N2A, SMAD4, are warranted to elucidate the mechanisms by which statin could impact tumor’s growth, invasion and metastasis.

Despite several strengths of the SEER-Medicare data, including a comparatively large sample size, generalizability to the U.S. population and detailed information on prescriptions, our study was limited by the lack of laboratory data on cholesterol, triglyceride, and glucose levels that would have informed the extent of metabolic disturbances in the population. Compared to pancreatic cancer patients exposed to low intensity statins, high intensity statin users were more likely to have obesity, dyslipidemia and diabetes, indicating more severe metabolic disturbances in this population; thus having laboratory-based data could have reduced residual confounding by severity of metabolic disease. We also lacked more granular data on cancer progression, which could have confounded the association between statin use and death, given that statin treatment may be withheld or discontinued in patients with short expected survival time. When we adjusted for such reverse causation by incorporating a lagged statin variable that effectively excludes the 2 months exposure period before death, the statin effect on survival was greatly attenuated. This demonstrates that it is important to consider how prescription drug treatment after cancer diagnosis is influenced by poor prognosis. Even with the adjustment for reverse causation, there still remains a possibility that the patients with severe symptoms and worse prognosis were unable to fill their prescriptions, or that statin was withheld in persons with metastases to the liver due to risk of transaminitis [22]. However, we saw the effect of statin treatment in those with low grade, resected cancers, who likely experienced less severe symptoms and faced a better prognosis. We lacked individual-level data on the socioeconomic status of cancer patients, which could have confounded the relationship between statin use and death. This is critical to consider, given that socioeconomically disadvantaged patients are less likely to take statin [23]. Because we restricted the population to those continuously enrolled in Medicare Parts B and D, which require monthly premiums, we believe that statin users and non-users would be comparable with respect to access to prescription drugs.

In conclusion, we found that statin treatment is associated with enhanced survival in patients with pancreatic adenocarcinoma. Residual confounding by factors related to healthy behavior, statin use, and survivorship, however, could not be excluded due to limitations of the claims-based data. Furthermore, our SEER-Medicare analysis was limited to elderly patients enrolled in Medicare, who are less likely to be offered surgery or more likely to refuse surgery compared to a younger population enrolled in private insurance companies [11]. This suboptimal management, combined with greater comorbid conditions, would have led to an even shorter survival than in a younger population, which limits the generalizability of the study.

In light of the potential benefits of statin and its widespread use, a clinical trial of statin in pancreatic cancers is warranted in those with resected, low-grade pancreatic cancer. If proven beneficial, statin could be a novel and readily implementable cancer intervention for low-grade, resectable pancreatic cancer types for which treatment options are few.
