# Correction: The Association of Statin Use after Cancer Diagnosis with Survival in Pancreatic Cancer Patients: A SEER-Medicare Analysis

**DOI:** 10.1371/journal.pone.0128730

**Published:** 2015-05-27

**Authors:** Christie Y. Jeon, Stephen J. Pandol, Bechien Wu, Galen Cook-Wiens, Roberta A. Gottlieb, C. Noel Bairey Merz, Marc T. Goodman

The sixth author’s name is spelled incorrectly. The correct name is: C. Noel Bairey Merz. The correct citation is: Jeon CY, Pandol SJ, Wu B, Cook-Wiens G, Gottlieb RA, Bairey Merz CN, et al. (2015) The Association of Statin Use after Cancer Diagnosis with Survival in Pancreatic Cancer Patients: A SEER-Medicare Analysis. PLoS ONE 10(4): e0121783. doi:10.1371/journal.pone.0121783

## References

[1] Jeon CY, Pandol SJ, Wu B, Cook-Wiens G, Gottlieb RA, Merz NB, et al (2015) The Association of Statin Use after Cancer Diagnosis with Survival in Pancreatic Cancer Patients: A SEER-Medicare Analysis. PLoS ONE 10(4): e0121783 doi: 10.1371/journal.pone.0121783 25830309 10.1371/journal.pone.0121783 PMC4382214

